# Niche-specific maize microbiomes enhance productivity and nitrogen uptake under intercropping

**DOI:** 10.3389/fmicb.2025.1711988

**Published:** 2026-01-15

**Authors:** Yao Chang, Jian Wang, Chengbin Xu, Fangying Qu, Xuekai Sun, Zhi Quan, Liming Yin, Yunting Fang, Chao Wang

**Affiliations:** 1 College of Environmental Science, Liaoning University, Shenyang, China; 2 CAS Key Laboratory of Forest Ecology and Silviculture, Institute of Applied Ecology, Chinese Academy of Sciences, Shenyang, China; 3 University of Chinese Academy of Sciences, Beijing, China

**Keywords:** intercropping, maize microbiome, plant-microbe interaction, maize biomass, nitrogen uptake

## Abstract

Intercropping is widely used to improve crop yield, but the microbial mechanisms driving biomass and nitrogen (N) gains remain unclear. In a maize-soybean intercropping system, we compared intercropped and monocultured maize to investigate niche-specific microbial processes. At the tasseling stage, bacterial and fungal communities were profiled across above- and belowground maize compartments and linked to organ-level biomass and N content. We found that intercropping significantly enhanced maize total biomass and nitrogen uptake, due to the greater increase in roots and leaves. The intercropping also restructured bacterial and fungal communities in a niche-specific manner: bacterial diversity declined in the phylloplane and root, leaf endospheres, whereas fungal diversity increased in the leaf endosphere and stem episphere. Moreover, higher bacterial diversity was associated with lower biomass and N content, while higher fungal diversity showed the opposite trend. Shifts in microbial composition, particularly enrichment of Proteobacteria, Bacteroidota, and Ascomycota, better predicted plant performance than diversity metrics alone. Overall, our findings suggest that intercropping enhances maize growth and N acquisition by steering niche-specific microbial assemblies, highlighting cross-compartment microbiome organization as a promising target for microbiome-informed crop management.

## Introduction

Maize (*Zea mays* L.) is one of the most widely cultivated staple crops worldwide, but it is often grown in monoculture systems that rely heavily on water and fertilizer inputs to sustain high yields (Nuss and Tanumihardjo, 2010; Tilman et al., 2011). Such input-intensive practices, however, pose environmental risks, including soil compaction, greenhouse gas emissions, and biodiversity loss (Lal, 1997; Dendooven et al., 2012). In contrast, legume-cereal intercropping improves land-use efficiency by facilitating temporal and spatial complementarity in resource utilization, while biological nitrogen fixation by legumes enhances soil nitrogen availability and reduces reliance on chemical fertilizers (Clark and Francis, 1985; Good et al., 2004; Bedoussac et al., 2015; You et al., 2023). These processes promote nitrogen uptake by maize and contribute to yield stability and ecological sustainability (Jensen et al., 2020; Bamboriya et al., 2022; Lai et al., 2022). Specifically, maize-soybean intercropping system enhances nitrogen homeostasis and nitrogen use efficiency (NUE) in maize through targeted regulation of nitrogen assimilation enzymes and optimizing soil–plant nitrogen cycling, which collectively enhance nitrogen uptake, utilization efficiency, and grain yield (Dang et al., 2020; Nasar et al., 2022). Some studies showed that maize-soybean intercropping increased total soil nitrogen by 14.5% and maize yield by 31.17% (Zhang et al., 2024). Additionally, nitrogen uptake in maize grains can increase by ~30% compared to monoculture systems (Nasar et al., 2022). Despite these benefits, most previous studies have primarily focused on crop performance and soil nutrient status, while the underlying biological mechanisms, particularly the role of the maize-associated microbiome in mediating nitrogen use efficiency, remain insufficiently understood.

Shifts in nitrogen-acquiring microbial communities play a central role in regulating soil nitrogen availability and plant uptake, with cascading consequences for crop yield (Tilman, 2020; Jing et al., 2022). Legume-maize intercropping enhance nutrient availability in the rhizosphere and improve both the diversity and abundance of rhizosphere microbial communities (Li et al., 2014; Wang et al., 2024). Increasing evidence indicates that intercropping with legumes improves the functional diversity of the rhizosphere microbiome, with stronger responses observed in fungal than in bacterial assemblages (Li et al., 2012; Qiao et al., 2024). In addition, intercropping maize or sorghum with soybean alters the rhizosphere soil microenvironment through interspecific root interactions, reshapes microbial community structure, accelerates the deposition of available nutrients, and ultimately enhances nutrient uptake and crop yield (Jiang et al., 2024a; Shao et al., 2025). Furthermore, intercropping increases the abundance of beneficial microorganisms while reducing the annotated abundance of potential pathogenic bacteria, thereby suppressing harmful microbes (Ansari et al., 2024; Jiang et al., 2024b). Moreover, synergistic interactions between rhizobia and arbuscular mycorrhizal fungi further enhance nutrient acquisition and yield (Meng et al., 2015; Zhang et al., 2020). Nevertheless, most current research remains restricted to rhizosphere microbial composition and potential function, and the broader contributions of maize-associated microbiomes to nitrogen use efficiency and productivity are still unclear.

Microbial communities form a dynamic continuum from the soil to the leaves, linking belowground root systems with aboveground plant organs and playing key roles in nutrient cycling and maintaining plant-soil stability (Vorholt, 2012; Trivedi et al., 2021; Jing et al., 2022; Compant et al., 2025). For example, peanut intercropping significantly increased bulk soil bacterial richness (Jiang et al., 2022). But, the diversity of key microbial taxa, rather than that of the overall microbial community, played a crucial role in maintaining ecosystem functioning (Zhao et al., 2022). Furthermore, studies have revealed that core microorganisms exert both direct and indirect influences on microbiome assembly, consequently playing a significant role in mediating host-microbiome interactions (Trivedi et al., 2021). Intercropping restructures fungal communities by altering dominant taxa composition and enhances mulberry-associated fungal diversity, thereby improving soil nutrient utilization to meet the growth demands of both mulberry and alfalfa (Zhang et al., 2019). Furthermore, practices such as straw return in intercropping systems can modulate microbial activities to reduce soil acidity, increase nutrient availability, and enrich functional bacterial taxa, thereby improving soil fertility and mitigating non-point source pollution (Cui et al., 2024; Wang et al., 2025). Collectively, these findings highlight the central role of plant-associated microbiomes in facilitating nitrogen acquisition and promoting crop productivity (Zhang et al., 2019; Hartmann and Six, 2023). While the rhizosphere has been extensively studied, the contribution of microbiomes associated with aboveground compartments (e.g., stems and leaves) to plant performance has received far less attention. Unlike belowground microbiota that are directly involved in nutrient acquisition, aboveground microbes may influence crop productivity indirectly, through modulation of systemic signaling, stress responses, or internal nutrient redistribution (Nadarajah and Rahman, 2021). Recent studies suggest that plant compartments harbor distinct, niche-specific microbial communities shaped by developmental stage, environmental conditions, and cropping practices (Xiong et al., 2021a). However, it remains unclear whether intercropping alters the structure and function of aboveground maize-associated microbiomes, or how such changes influence nitrogen dynamics and yield formation.

In this study, we employed high-throughput sequencing to investigate the maize microbiome at the tasseling stage in a maize-soybean intercropping system. We conducted a comprehensive analysis of microbial communities across eight distinct ecological niches, including the aboveground niches (phylloplane, leaf endosphere, stem episphere, stem endosphere) and belowground niches (rhizosplane, root endosphere, rhizosphere soil, and bulk soil). Our objectives were to elucidate the compositional features of maize-associated microbial communities under intercropping conditions and to explore their potential relationships with nitrogen uptake and yield formation. We hypothesized that: (1) intercropping reshapes the microbial community structure across multiple maize-associated niches, particularly in aboveground tissues such as stems and leaves; and (2) changes in aboveground microbiota are associated with plant nitrogen use and crop yield. To test these hypotheses, we compared microbial community composition and diversity between monocropping and intercropping systems across different plant niches. Our study provides new insights into maize microbiome assembly under intercropping systems and offers microbiome-informed strategies to promote sustainable crop production.

## Materials and methods

### Field trial and treatments

The field experiment was established in spring 2022 in Changtu (123°58′E, 42°48′N), Liaoning Province, Northeastern China. Before the experiment, surface soil (0–20 cm) was collected to determine the background properties. The soil had a pH of 5.1, total carbon content of 11.94 g kg^−1^, total nitrogen (TN) of 11.43 g kg^−1^, total phosphorus (P) of 0.47 g kg^−1^, nitrate nitrogen (NO_3_^−^-N) of 21.90 mg kg^−1^, and ammonium nitrogen (NH_4_^+^-N) of 6.28 mg kg^−1^.

A randomized block design was adopted with two treatments (monocropping and intercropping), each consisting of three biological replicates (*n* = 3) using 40 × 40 m^2^ plots, resulting in a total of six samples (*n* = 6). Two cropping systems were compared: (1) maize monoculture (MM) and (2) maize-soybean intercropping (IM), in which six rows of maize alternated with four rows of soybean (6M:4S). Maize and soybean rows were spatially arranged in alternating strips, with maize planted at 23 cm within rows and 58 cm between rows, and soybean planted at 15 cm within rows and 65 cm between rows. The same maize cultivar and soybean variety were used in both monoculture and intercropping systems. Plant density was maintained at 60,000 plants ha^−1^ for maize and 300,000 plants ha^−1^ for soybean, and both plant density and spacing were kept consistent across treatments to ensure comparability. Fertilizers were applied as urea (225 kg N ha^−1^), diammonium phosphate (60 kg P_2_O_5_ ha^−1^), and potassium sulfate (60 kg K_2_O ha^−1^). Soil moisture and temperature were not continuously monitored during the growing season. All plots were managed under the same field conditions without artificial control of environmental variables.

### Soil and maize plant sampling

Plant and soil samples were collected at the tasseling stage of maize in July 2022. Bulk soil was collected from three randomly selected points within each plot. Similarly, three maize plants were randomly selected for organ and rhizosphere sampling. For aboveground tissues, one to two leaves from the mid-upper section and stem segments from the corresponding position were collected and immediately placed on ice. For belowground samples, roots were excavated with minimal disturbance, and loosely attached soil was removed as bulk soil. The soil tightly adhering to root surfaces was brushed off and designated as rhizosphere soil. All soil samples were homogenized, passed through a 20-mm mesh sieve, and stored at 4 °C for subsequent analyses. The plant tissues were oven-dried at 70 °C for 48 h to a constant weight for subsequent biomass and plant nitrogen content determination. Nitrogen concentration was determined using an elemental analyzer (Elementar Vario MICRO cube, Hanau, Germany) on approximately 3 mg of soil or 15 mg of dried, ground plant tissue.

### DNA extraction and bioinformatics analysis

Total DNA from soil samples was extracted from 0.4 g of soil using the MoBio PowerSoil DNA Extraction Kit (MoBio Laboratories, USA). Epiphytic microbes on plant epispheres were collected by vortexing 10–15 g of leaves, and 3–5 g of root samples in PBS buffer (130 mM NaCl, 7 mM Na_2_HPO_4_, 3 mM NaH_2_PO_4_, pH = 7.4), followed by DNA extraction using the same PowerSoil kit. For endophytic communities, plant tissues were surface-sterilized (70% ethanol for 1 min, 3% sodium hypochlorite for 3 min, and five washes with sterile water) before DNA isolation.

Bacterial community analysis targeted the V5-V7 region of the 16S rRNA gene using two primer pairs: 799F (5′-AACMGGATTAGATACCCKG-3′)/1392R (5′-ACGGGCGGTGTGIRC-3′) and 799F/1193R (5′-ACGTCATCCCCACCTTCC-3′) (Chelius and Triplett, 2001; Horton et al., 2014; Tan et al., 2022). Fungal communities were analyzed by amplifying the ITS1 region using ITS1F (5′-CTTGGTCATTTAGAGGAAGTAA-3′) and ITS2R (5′-GCTGCGTTCTTCATCGATGC-3′) primers (Schoch et al., 2012). PCR products were purified and subjected to paired-end sequencing (2 × 300 bp) on the Illumina MiSeq platform (MiSeq Reagent Kit v3). Raw reads were processed using the DADA2 pipeline (Callahan et al., 2016). For bacterial 16S rRNA gene sequences, reads were quality filtered and trimmed [truncLen = c(280, 260), maxEE = c(2, 2), truncQ = 2] to remove low-quality bases before dereplication, denoising, and chimera removal to infer exact amplicon sequence variants (ASVs). For fungal ITS reads, quality filtering and trimming parameters were adjusted according to read quality profiles [typically truncLen = c(250, 200)], given the variable length of ITS regions. Forward and reverse reads were merged, and chimeric sequences were removed to generate high-confidence ASVs. Sequences classified as chloroplasts or mitochondria were excluded to retain only bacterial ASVs for downstream analyses. Taxonomic assignment of ASVs was performed using the SILVA database (http://www.arb-silva.de, version 138.1) for bacteria and the UNITE database (https://unite.ut.ee, version 9.0) for fungi with a minimum bootstrap confidence threshold of 50 and 80%, respectively. Microbial alpha diversity (Shannon index) was calculated based on species abundance data. Beta diversity was calculated using Bray–Curtis dissimilarities, and differences in community composition among samples were visualized through Principal Coordinate Analysis (PCoA). Raw sequencing data have been deposited in the Science Data Bank via the following link: https://www.scidb.cn/s/NjEJFf.

### Statistical analysis

Statistical analyses and data visualization of biomass and nitrogen concentration were conducted using R software (v4.4.1). Independent-samples *t*-tests were performed to compare differences in biomass and nitrogen content between monoculture (MM) and intercropping (IM) treatments across plant organs (root, stem, leaf, and whole plant), with three biological replicates per treatment. Data are presented as mean ± standard error. A two-way analysis of variance (ANOVA) was conducted to evaluate the effects of treatment and plant niche on microbial alpha diversity, followed by Tukey’s HSD post-hoc tests where appropriate. Pairwise Student’s *t*-tests were further applied to compare alpha diversity indices between MM and IM within each niche. Microbial beta-diversity based on Bray–Curtis dissimilarity was assessed using permutational multivariate analysis of variance (PERMANOVA, 999 permutations) to test the effects of treatment, niche, and their interaction on community composition. The assumption of homogeneity of multivariate dispersions was verified prior to PERMANOVA. To confirm the robustness of community-level differences, analysis of similarities (ANOSIM) was also performed, yielding consistent results with PERMANOVA. Differences in the relative abundance of dominant genera between cropping systems were examined for the top 20 most abundant genera. Within each niche, two-sided Student’s *t*-tests were used to identify genera showing significant differences (*p* < 0.05) between MM and IM. Negative controls were included during DNA extraction and PCR amplification, and no contamination was detected. Pearson’s correlation tests were performed to assess the relationships between Shannon-based alpha diversity, PCoA1-based beta diversity, and maize biomass and nitrogen content, as well as the correlations between individual taxa across aboveground and belowground niches and maize biomass and nitrogen content. Statistical significance was determined at *p* < 0.05. Representative sequences of significantly associated ASVs (*p* < 0.05) were aligned and used to construct maximum likelihood phylogenetic trees in IQ-TREE. Trees were visualized in R using ggtree (v3.12.0) and ggtreeExtra (v1.14.0), and annotated with Pearson correlation heatmaps and niche distribution matrices to illustrate organ-specific associations and compartmentalization. The R packages vegan (v2.6-8) and ggplot2 (v3.5.1) were primarily used for ecological and graphical analyses, Hmisc (v4.7-0) for Pearson’s correlation tests, and pheatmap (v1.0.12) for data processing.

## Result

### Maize biomass and nitrogen content

**Figure 1:**
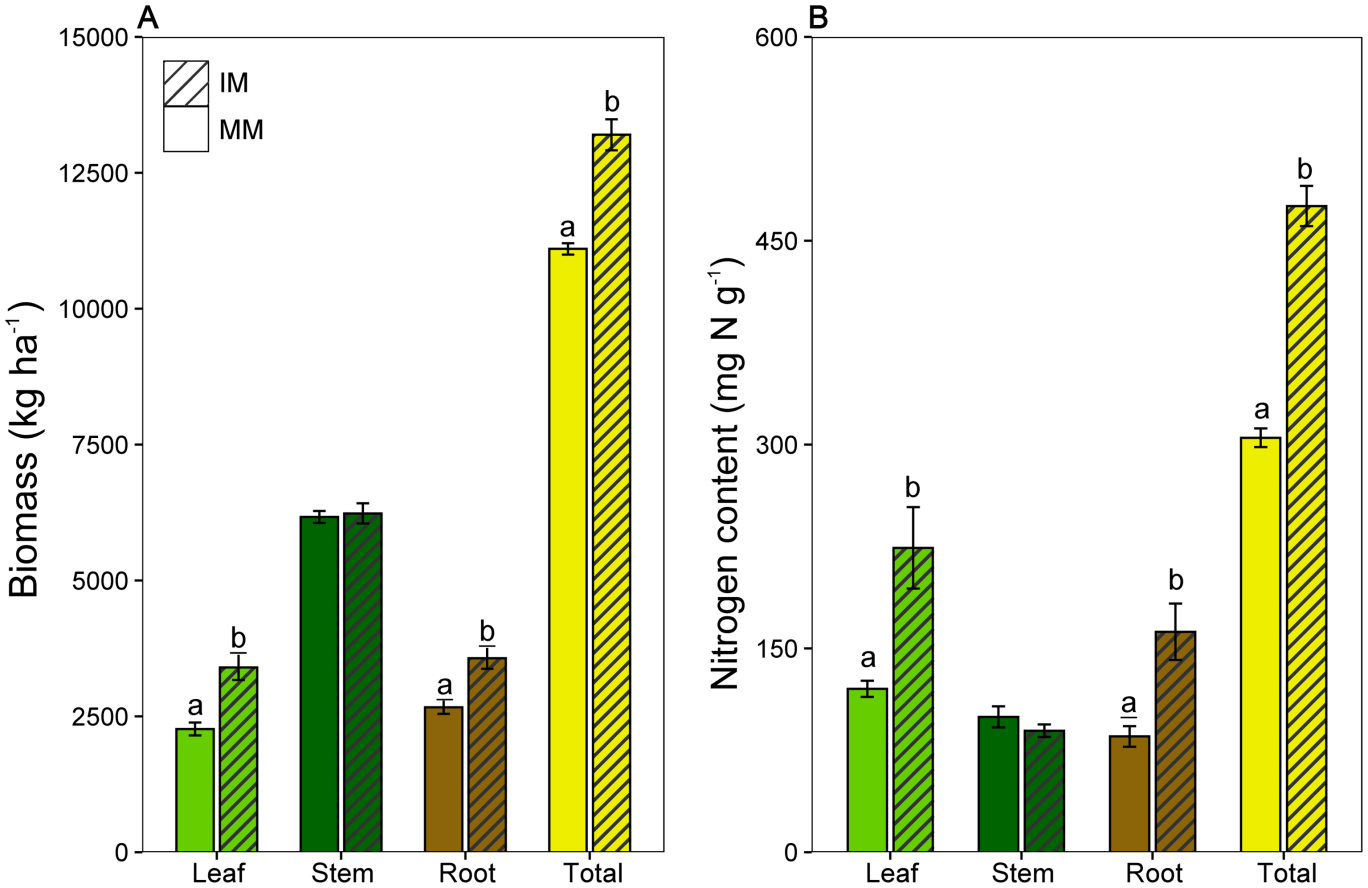
Plant biomass and nitrogen content of maize grown under monoculture (MM) and intercropping (IM) systems. **(A)** Biomass (kg ha^−1^) of leaf, stem, root, and total plant. **(B)** Nitrogen content (mg N g^−1^) in individual organs and total plant. Different lowercase letters indicate significant differences between treatments (*p* < 0.05; one-way ANOVA with Tukey’s HSD test).

At the tasseling stage, intercropping significant altered biomass allocation relative to monocropping (Figure 1A, *p* < 0.05). Total biomass was higher under intercropping (222.00 ± 3.61 kg ha^−1^) than monocropping (264.00 ± 9.85 kg ha^−1^). Intercropping increased root and leaf biomass in maize, while stem biomass showed no significant difference between treatments. Consistently, intercropping significantly increased nitrogen content in both the root and leaf tissues compared to monocropping (Figure 1B, *p* < 0.05). Specifically, root nitrogen content was significantly higher in intercropping (123.52 ± 36.00 mg g^−1^) compared to monocropping (75.92 ± 13.09 mg g^−1^), and leaf nitrogen content was also significantly higher in intercropping (194.74 ± 80.58 mg g^−1^) compared to monocropping (122.76 ± 10.34 mg g^−1^).

### Microbial community diversity

**Figure 2:**
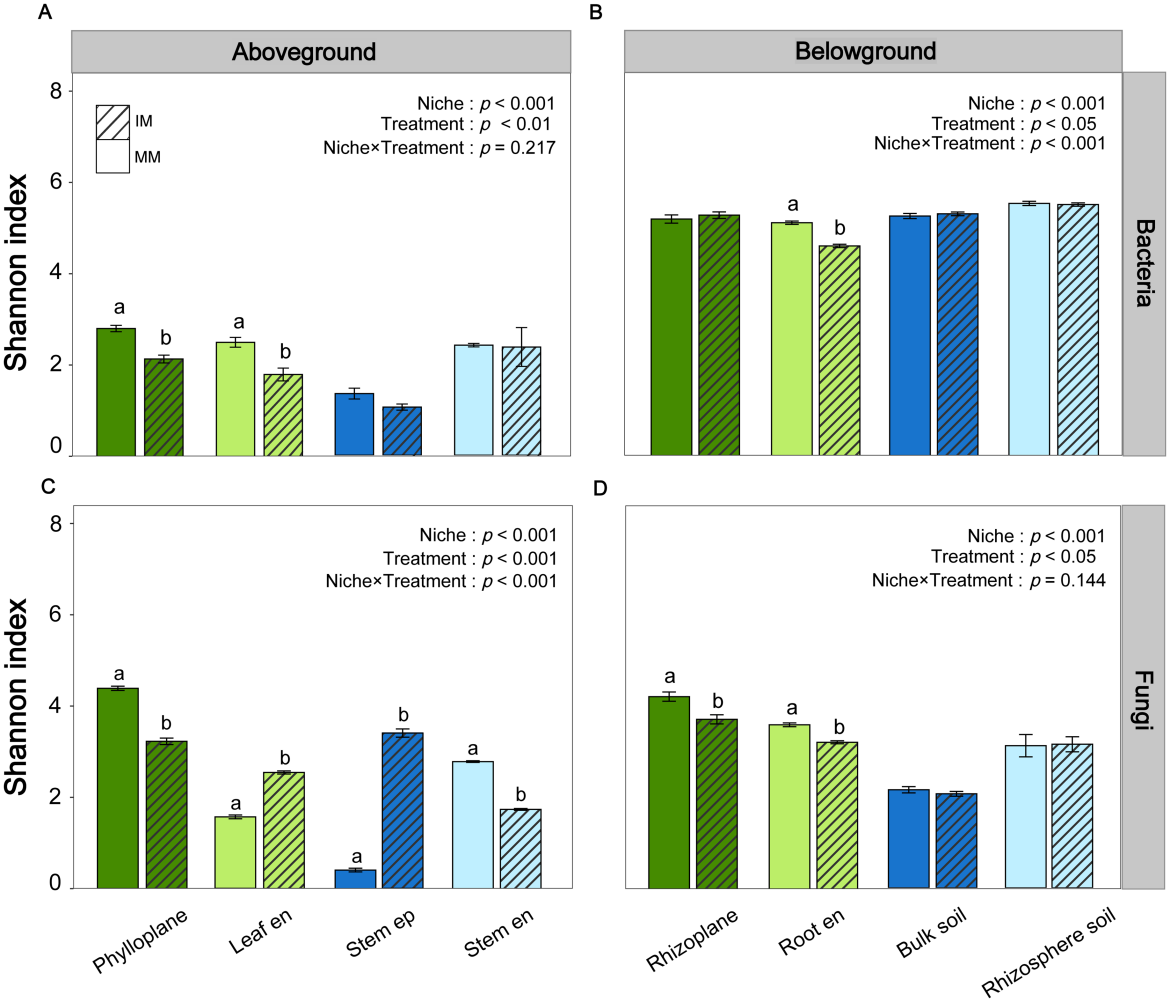
Shannon diversity index of bacterial and fungal communities in maize grown under monoculture (MM) and intercropping (IM) systems. **(A,B)** Bacterial communities in above- and belowground niche. **(C,D)** Fungal communities in above- and belowground niche. Aboveground niches include the phylloplane, leaf endosphere (Leaf en), stem episphere (Stem ep), and stem endosphere (Stem en), while the belowground niches include the rhizoplane, root endosphere (Root en), bulk soil, and rhizosphere soil. Different lowercase letters denote significant differences among treatments, niches, and their interactions (*p* < 0.05; two-way ANOVA with Tukey’s HSD test).

**Figure 3:**
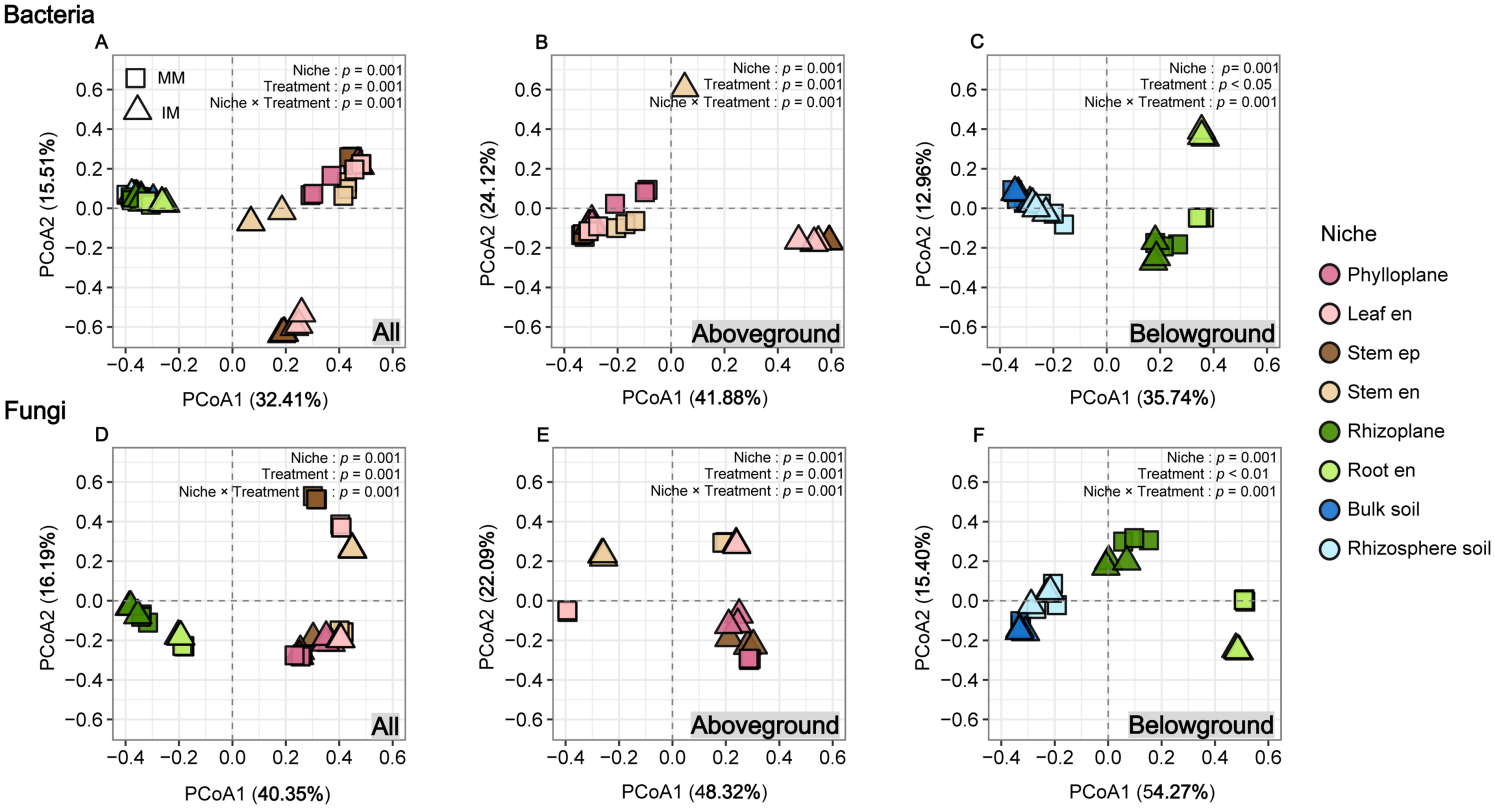
Principal coordinate analysis (PCoA) of bacterial and fungal communities based on Bray-Curtis dissimilarity in maize grown under monoculture and intercropping systems. Bacterial communities across **(A)** all niche, **(B)** aboveground niches, and **(C)** belowground niches, respectively. Fungal communities across **(D)** all niche, **(E)** aboveground niches, and **(F)** belowground niches, respectively. Aboveground niches include the phylloplane, leaf endosphere (Leaf en), stem episphere (Stem ep), and stem endosphere (Stem en), while the belowground niches include the rhizoplane, root endosphere (Root en), bulk soil, and rhizosphere soil.

Alpha and beta diversity analyses revealed niche-specific and contrasting responses of bacterial and fungal communities to intercropping (Figures 2, 3). For bacteria, Shannon diversity decreased under intercropping in the phylloplane, leaf endosphere, and root endosphere, indicating reduced alpha diversity in these niches (Figures 2A,B; *p* < 0.05). For fungi, diversity responses were niche- and treatment-dependent (*p* < 0.001), with intercropping reducing diversity in the phylloplane and stem endosphere but increasing it in the leaf endosphere and stem episphere (Figure 2C). At belowground, intercropping significantly reduced fungal diversity in the rhizoplane and root endosphere relative to monocropping (Figure 2D; *p* ≤ 0.05). PCoA showed a significant separation of bacterial and fungal communities between aboveground and belowground compartments (PERMANOVA, *p* ≤ 0.05; Figures 3A,D), and between cropping systems within each stratum for bacteria (Figures 3B,C) and fungi (Figures 3E,F).

### Microbial community composition

**Figure 4:**
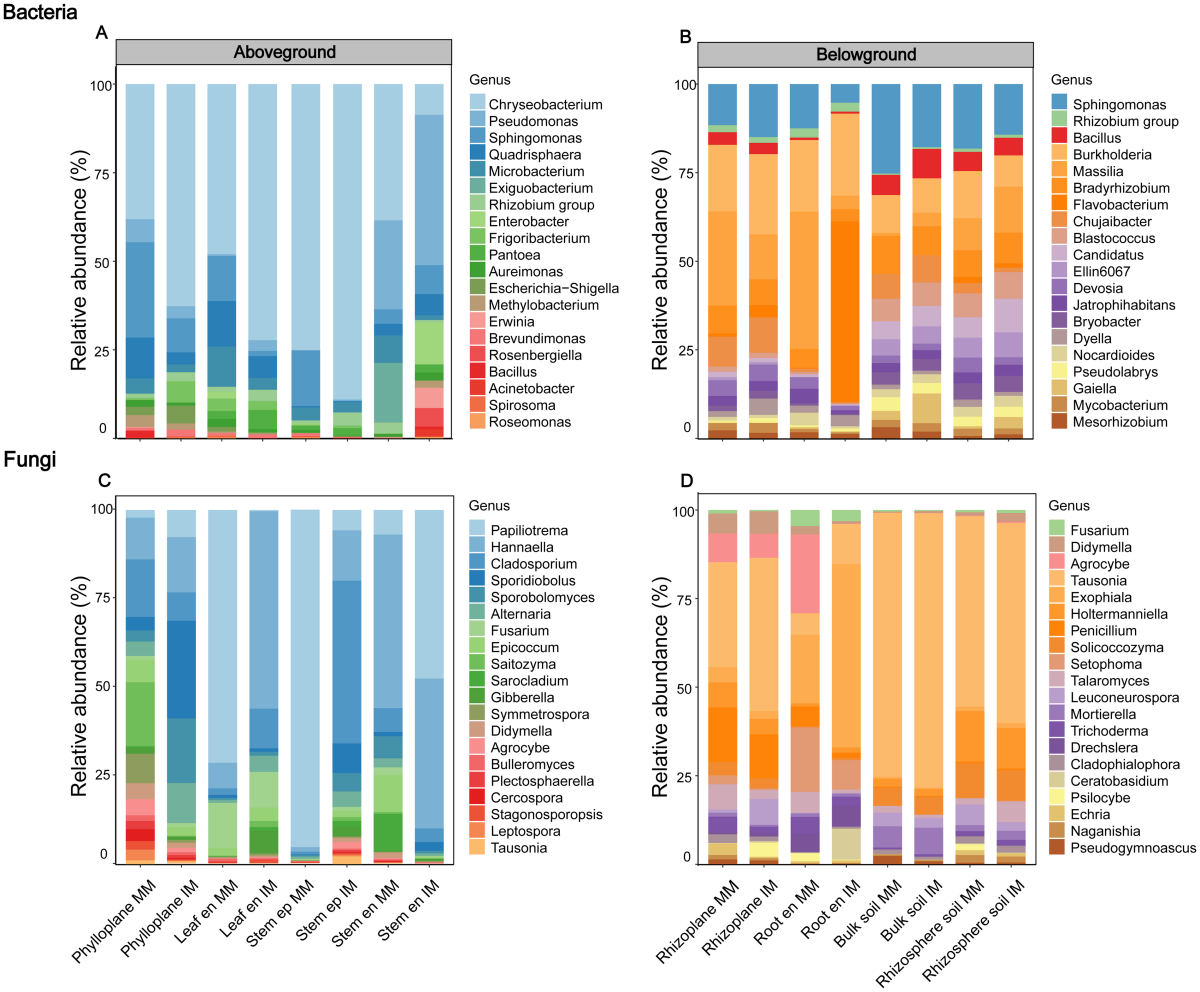
Relative abundance of the top 20 bacterial and fungal genera across aboveground and belowground niches in maize grown under monoculture (MM) and intercropping (IM) systems. **(A)** Bacterial community’s aboveground niches, **(B)** bacterial community’s belowground niches, **(C)** fungal community’s aboveground niches, **(D)** fungalcommunity’s belowground niches. Aboveground niches include the phylloplane, leaf endosphere (Leaf en), stem episphere (Stem ep), and stem endosphere (Stem en), while the belowground niches include the rhizoplane, root endosphere (Root en), bulk soil, and rhizosphere soil.

At the genus level, intercropping and monoculture systems fostered compositionally distinct microbial communities in both above- and belowground niches of maize, with differential abundance analysis confirming the key indicator taxa that distinguished the two systems (Figure 4; Table 1).

**Table 1:** Genera enriched under intercropping (IM) relative to monoculture (MM) across maize niches, derived from the top 20 bacterial and fungal genera.

Type	Niche	Phylum	Genus	Significance
Bacteria	Leaf endosphere	Proteobacteria	*Pseudomonas*	*p* < 0.05
		Proteobacteria	Rhizobium group*	*p* < 0.05
		Bacteroidota	*Chryseobacterium*	*p* < 0.05
	Phylloplane	Actinobacteriota	*Frigoribacterium*	*p* < 0.001
	Stem endosphere	Bacteroidota	*Chryseobacterium*	*p* < 0.01
		Bacteroidota	Rhizobium group*	*p* < 0.05
		Firmicutes	*Bacillus*	*p* < 0.05
		Proteobacteria	*Pantoea*	*p* < 0.05
	Stem episphere	Bacteroidota	Rhizobium group*	*p* < 0.05
		Actinobacteriota	*Frigoribacterium*	*p* < 0.05
	Root endosphere	Bacteroidota	*Flavobacterium*	*p* < 0.01
		Actinobacteriota	*Blastococcus*	*p* < 0.01
	Rhizoplane	Proteobacteria	Burkholderia group*	*p* < 0.05
Fungi	Leaf endosphere	Basidiomycota	*Hannaella*	*p* < 0.001
		Ascomycota	*Cladosporium*	*p* < 0.01
	Phylloplane	Ascomycota	*Cladosporium*	*p* < 0.001
	Stem episphere	Basidiomycota	*Hannaella*	*p* < 0.001
		Ascomycota	*Cladosporium*	*p* < 0.01
	Stem endosphere	Ascomycota	*Cladosporium*	*p* < 0.05
	Root endosphere	Basidiomycota	*Ceratobasidium*	*p* < 0.001
		Ascomycota	*Exophiala*	*p* < 0.001
		Basidiomycota	*Psilocybe*	*p* < 0.01
		Basidiomycota	*Tausonia*	*p* < 0.05
	Rhizoplane	Basidiomycota	*Tausonia*	*p* < 0.01
		Basidiomycota	*Ceratobasidium*	*p* < 0.05
		Ascomycota	*Leuconeurospora*	*p* < 0.05
		Basidiomycota	*Psilocybe*	*p* < 0.05

Aboveground niches include the phylloplane, leaf endosphere (Leaf en), stem episphere (Stem ep), and stem endosphere (Stem en), while the belowground niches include the rhizoplane, root endosphere (Root en), bulk soil, and rhizosphere soil.

*Rhizobium group refers to the combined genera *Allorhizobium, Neorhizobium, Pararhizobium*, and *Rhizobium*; *Burkholderia group* refers to combined genera *Burkholderia*, *Caballeronia* and *Paraburkholderia*.

For bacteria, the divergence was most pronounced in endophytic compartments. In addition, the intercropping root endosphere was strongly enriched for *Flavobacterium* (*p* < 0.01), whereas monoculture plants retained higher levels of *Massilia*. At aboveground, intercropping was distinguished by the enrichment of key genera, including *Pantoea* and *Bacillus* in the stem endosphere (*p* < 0.05), and *Frigoribacterium* (*p* < 0.001) in the phylloplane.

The differentiation in fungal communities was even more pronounced (Figure 4). At belowground, the divergence culminated in the root endosphere, where the intercropping microbiome was dominated by *Exophiala* and *Ceratobasidium* (*p* < 0.001), while the monoculture community was defined by a completely different set of genera, including *Agrocybe*, *Trichoderma*, and *Mortierella*. At aboveground, the phylloplane was dominated by *Papiliotrema* in monoculture but by *Cladosporium* (*p* < 0.001) in intercropping. *Hannaella* (*p* < 0.001) also served as a key indicator for intercropping in stem episphere.

### Links between microbial properties and plant biomass and nitrogen

**Figure 5:**
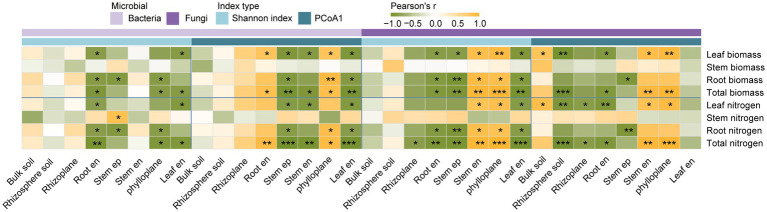
Pearson correlation coefficients between microbial (bacteria and fungi) communities and maize biomass and nitrogen content. Yellow and green indicate positive and negative correlations, respectively, with asterisks denoting significance levels (***p < 0.001; **p < 0.01; *p < 0.05). The Shannon index represents the alpha diversity of bacterial and fungal communities. Bacterial PCoA1 and fungal PCoA1 correspond to the first-dimensional PCoA ordination from Bray-Curtis PCoA based on bacterial and fungal taxonomic composition, respectively. Aboveground niches include the phylloplane, leaf endosphere (Leaf en), stem episphere (Stem ep), and stem endosphere (Stem en), while the belowground niches include the rhizoplane, root endosphere (Root en), bulk soil, and rhizosphere soil.

For bacteria, Shannon diversity in aboveground niches was generally negatively related to plant properties (Figure 5). For example, higher diversity in the stem episphere correlated with reduced leaf biomass (*p* < 0.01), while leaf endophytic diversity correlated negatively with total N (*p* < 0.001). Besides, root endosphere diversity was negatively associated with total biomass. Consistently, bacterial community composition PCoA1 was negatively correlated with total nitrogen in the stem episphere (*p* < 0.001) and with total biomass in phylloplane (*p* < 0.01), but positively associated with biomass in the phylloplane (*p* < 0.01) and root endophytes (*p* < 0.05).

In contrast, fungal diversity exhibited more positive relationships with plant traits (Figure 5). Phylloplane fungal diversity was positively associated with plant total biomass (*p* < 0.01), wheres stem episphere diversity was negatively associated with leaf nitrogen (*p* < 0.05). Fungal community composition further exhibited clear compartment specificity, as phylloplane PCoA1 correlated positively with total nitrogen (*p* < 0.01), whereas rhizosphere PCoA1 correlated negatively with biomass (*p* < 0.05).

### Links between microbial taxa and plant biomass and nitrogen

**Figure 6:**
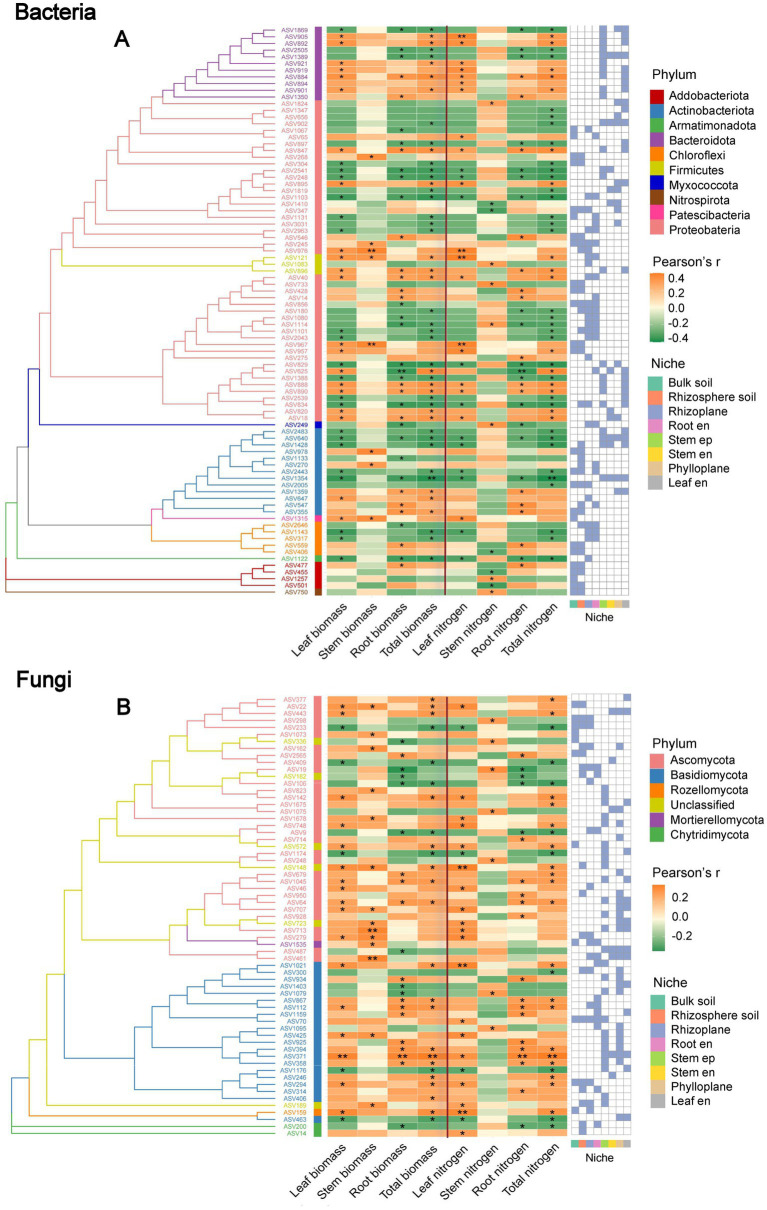
Phylogenetic relationships of microbial taxa associated with maize biomass and nitrogen content. Phylogenetic trees of species-level ASVs are shown for bacterial **(A)** and fungal **(B)** communities. The accompanying heatmaps display Pearson correlation coefficients between microbial taxa and biomass or nitrogen content across different maize organs. Yellow and green indicate positive and negative correlations, respectively, with asterisks denoting significance levels (***p < 0.001; **p < 0.01; *p < 0.05). The right panel shows the niche-specific distribution of microbial taxa, with blue indicating presence and white indicating absence. Aboveground niches include the phylloplane, leaf endosphere (Leaf en), stem episphere (Stem ep), and stem endosphere (Stem en), while the belowground niches include the rhizoplane, root endosphere (Root en), bulk soil, and rhizosphere soil.

Among bacteria, 85 ASVs were significantly correlated with maize biomass and nitrogen content (Figure 6A). *Proteobacteria* accounted for >50% of these ASVs, followed by *Acidobacteriota* and *Bacteroidota* (~15% each). *Bacteroidota* were exclusively found in aboveground niches and showed mixed associations: generally negative with root biomass and N, but positive with those of leaf. *Proteobacteria* and *Acidobacteriota*, showing predominantly positive correlations. For total biomass, associated ASVs were 55% *Proteobacteria*, while for total N the proportion of *Proteobacteria* increased to 61%. *Proteobacteria* and *Actinobacteriota* were widely distributed across the rhizosphere as well as belowground and aboveground plant microbiomes, with their associations with maize biomass and nitrogen varying among niches.

In fungi, 63 ASVs were significantly associated with maize traits (Figure 6B), dominated by *Ascomycota* (>40%) and *Basidiomycota* (>20%). Overall, *Ascomycota* showed widespread positive correlations with biomass and N across both above- and below-ground niches, while a few ASVs were negatively linked with N in belowground tissues. By contrast, *Basidiomycota* from aboveground compartments were often negatively related to root and leaf biomass, though positively associated ASVs were also distributed across multiple niches.

## Discussion

Intercropping is a sustainable strategy that enhances resource use efficiency and crop productivity by promoting complementary interactions between plants and their microbiomes (Tilman, 2020; Jiang et al., 2024a). Our results showed that intercropping promoted maize biomass accumulation and nitrogen uptake, with pronounced effects in roots and leaves. These gains were accompanied by pronounced, niche-specific shifts in microbial diversity and composition across above- and belowground compartments, indicating that intercropping reshapes both soil-derived inputs and plant-associated microbiomes. Compartment-specific associations between microbial assemblages and maize nitrogen content further suggest that above- and belowground microbiota contribute differentially to host nutrient acquisition. Together, these findings provide a mechanistic framework linking microbiome restructuring to the intercropping advantage, setting the stage for a detailed examination of niche-specific microbial contributions to maize growth and nitrogen uptake.

Intercropping significantly increased maize biomass and nitrogen accumulation at the tasseling stage, particularly in roots and leaves, consistent with the well-documented agronomic benefits of this practice (Gao et al., 2010; Wang et al., 2023). These organ-specific gains suggest that intercropping alters nitrogen allocation among maize organs, contributing to higher total biomass and nitrogen accumulation. The principle that intercropping stimulates root activity and improves nitrogen use efficiency is well established in maize-legume systems, including maize-soybean intercropping (Zheng et al., 2021; Qiao et al., 2024; Guo et al., 2025). In contrast, stem biomass remained relatively stable, suggesting a differential allocation of resources among organs during critical developmental stages (Poorter et al., 2012; Xia et al., 2013). Importantly, these phenotypic patterns provide a foundation for our central hypothesis that the intercropping advantage is associated with distinct, niche-specific microbial communities.

At the tasseling stage, intercropping drove a pronounced, niche-specific reorganization of maize-associated bacterial and fungal communities from root to leaf, with clear differentiation between above and belowground compartments. While plant niche remained the primary determinant of community structure, intercropping acted as a secondary selective force that reduced aboveground bacterial diversity but induced more complex, niche-dependent fungal patterns, possibly reflecting host-mediated adaptations to organ-specific microenvironments (Agler et al., 2016; Xiong et al., 2021b). Intercropping induced a pronounced reorganization of maize-associated bacterial and fungal communities, extending beyond the rhizosphere to the entire plant holobiont. Previous intercropping studies have primarily emphasized rhizosphere processes that enhance nitrogen fixation and turnover (Stern, 1993; Qiao et al., 2024; Zhang et al., 2024). Our findings expand this view by showing that intercropping also reshapes above-ground microbiomes, suggesting their potential involvement in nutrient-related interactions. In the root endosphere, the enrichment of *Flavobacterium* under intercropping may indicate enhanced microbially mediated nutrient transformation, including potential contributions to nitrogen and phosphorus cycling, as well as phytohormone driven regulation of root development under stress conditions (Kwak et al., 2018; Lidbury et al., 2021; El Sabagh et al., 2022). Conversely, the above-ground enrichment of genera such as *Pantoea*, *Frigoribacterium*, and *Cladosporium* may reflect niche-specific adaptation and possible functional diversification of the maize microbiome during reproductive growth, as these taxa are known to contribute to nitrogen fixation, hormone regulation, and pathogen suppression, thereby enhancing plant growth and stress tolerance under variable environmental conditions (Rastogi et al., 2012; Qin et al., 2016; Li et al., 2023). Collectively, these findings suggest that intercropping drives a coordinated restructuring of the maize microbiome across niches, potentially enhancing nutrient use efficiency and biomass accumulation (Lidbury et al., 2021; Zheng et al., 2021; Qiao et al., 2024).

Niche-specific microbial diversity and community composition were tightly linked to maize biomass and nitrogen uptake, reflecting distinct contributions of bacterial and fungal assemblages. In aboveground compartments, higher bacterial alpha diversity was generally associated with reduced maize biomass and nitrogen content. This pattern may reflect functional redundancy, where multiple bacterial species perform overlapping roles such that adding more species does not enhance functional output, and can even generate competition for resources or space among microbes (Louca et al., 2018; Wicaksono et al., 2024). Such redundancy or competitive interactions may limit the positive contribution of microbial diversity to host nutrient acquisition and growth (Schlechter et al., 2023; Wicaksono et al., 2024). Functional predictions further support these patterns: in all belowground samples, bacteria associated with ureolysis were consistently abundant, especially in rhizosphere soil and roots, indicating that urea decomposition is an important nitrogen-cycling process. Other nitrogen-cycle functions, including nitrification, denitrification, and nitrate/nitrite reduction, were present but at lower or more variable abundances, while nitrogen-fixing bacteria were detected in small amounts. In contrast, aboveground compartments showed distinct functional profiles: nitrogen-fixing bacteria were significantly enriched in the stem endosphere and the phylloplane, and ureolysis was also present in leaves, suggesting the presence of functionally important diazotrophs aboveground. Fungal functional predictions revealed high abundances of saprotrophs and arbuscular mycorrhizal fungi in belowground niches, consistent with mutualistic associations enhancing nutrient uptake, while aboveground fungal communities were dominated by endophytes, with epiphytes also abundant; other saprotrophs were present but less dominant. These functional patterns are consistent with the contrasting correlations observed between bacterial and fungal diversity and maize performance, highlighting potential competition or redundancy among bacteria versus mutualistic benefits from fungi (Xiong et al., 2021a; Labouyrie et al., 2023; Almeida et al., 2024; Wang et al., 2026). Beta diversity patterns further indicated that shifts in community composition, rather than richness alone, shaped the ecological differentiation of maize microbiomes across niches (Agler et al., 2016; Xiong et al., 2021a). *Proteobacteria* dominated plant-associated taxa linked to biomass and nitrogen, consistent with their versatile roles in N, P, and Fe cycling, while *Acidobacteriota and Actinobacteriota* showed niche-specific associations, suggesting complementary contributions to carbon and nutrient turnover (Trivedi et al., 2021; Orellana et al., 2022); *Bacteroidota*, enriched aboveground, may support organic phosphorus mineralization and stress resilience (Lidbury et al., 2021; Pan et al., 2023). Among fungi, *Ascomycota* were positively correlated with biomass and nitrogen, potentially via hyphal networks and bioactive metabolites, whereas *Basidiomycota* exhibited mixed effects, possibly reflecting competition or antagonism (Challacombe et al., 2019; Gao et al., 2020). Overall, these results reveal that microbial alpha and beta diversity, along with community composition, are closely linked to maize biomass and nitrogen accumulation, highlighting the ecological relevance of niche-specific microbial assemblages, where *Proteobacteria*, *Bacteroidota* and *Ascomycota* form a core group associated with higher plant performance, and intercropping promotes a compositionally specialized microbiome adapted to distinct plant niches.

Our study highlights agronomic practical arising from intercropping-induced microbiome shifts. Tailored cropping designs or introduction of core microbial consortia can steer plant-associated microbiomes toward functionally optimized assemblages, enhancing nutrient cycling and crop productivity. Field studies show that rhizosphere inoculants can reshape native microbial communities and improve nutrient uptake (Xie et al., 2024; Francioli et al., 2025). Integrating targeted inoculation with intercropping layout, such as spatial–temporal design and inter-row spacing, may further boost these benefits. Future work using functional omics can elucidate mechanisms of key taxa and refine inoculant strategies for field application.

## Conclusion

Our study demonstrates that intercropping fundamentally reshapes the maize-associated microbiome across below- and aboveground compartments, forming distinct bacterial and fungal assemblages closely linked to plant biomass and nitrogen accumulation. Intercropping enhanced root and leaf growth and nitrogen content, accompanied by niche-specific shifts in microbial diversity that reflect functional strategies for nutrient acquisition. Overall, intercropping not only restructures soil–plant interactions but also drives coordinated reorganization of the plant holobiont, highlighting its potential for microbiome-informed management to optimize nutrient cycling and crop productivity in sustainable agriculture.

## Data Availability

The datasets presented in this study can be found in online repositories. The names of the repository/repositories and accession number(s) can be found in the article/Supplementary material.

## References

[ref1] Agler M. T. Ruhe J. Kroll S. Morhenn C. Kim S. Weigel D. . (2016). Microbial hub taxa link host and abiotic factors to plant microbiome variation. PLoS Biol. 14:e1002352. doi: 10.1371/journal.pbio.1002352, 26788878 PMC4720289

[ref2] Almeida B. K. Tran E. H. Afkhami M. E. (2024). Phyllosphere fungal diversity generates pervasive nonadditive effects on plant performance. New Phytol. 243, 2416–2429. doi: 10.1111/nph.19792, 38719779

[ref3] Ansari W. A. Kumar M. Krishna R. Singh S. Zeyad M. T. Tiwari P. . (2024). Influence of rice-wheat and sugarcane-wheat rotations on microbial diversity and plant growth promoting bacteria: insights from high-throughput sequencing and soil analysis. Microbiol. Res. 278:127533. doi: 10.1016/j.micres.2023.127533 37924641

[ref4] Bamboriya S. D. Bana R. S. Kuri B. R. Kumar V. Bamboriya S. D. Meena R. P. (2022). Achieving higher production from low inputs using synergistic crop interactions under maize-based polyculture systems. Environ. Sustain. 5, 145–159. doi: 10.1007/s42398-022-00228-7

[ref5] Bedoussac L. Journet E. P. Hauggaard Nielsen H. Naudin C. Corre Hellou G. Jensen E. S. . (2015). Ecological principles underlying the increase of productivity achieved by cereal-grain legume intercrops in organic farming. A review. Agron. Sustain. Dev. 35, 911–935. doi: 10.1007/s13593-014-0277-7

[ref6] Callahan B. J. McMurdie P. J. Rosen M. J. Han A. W. Johnson A. J. A. Holmes S. P. (2016). DADA2: high-resolution sample inference from Illumina amplicon data. Nat. Methods 13, 581–583. doi: 10.1038/nmeth.3869, 27214047 PMC4927377

[ref7] Challacombe J. F. Hesse C. N. Bramer L. M. McCue L. A. Lipton M. Purvine S. . (2019). Genomes and secretomes of Ascomycota fungi reveal diverse functions in plant biomass decomposition and pathogenesis. BMC Genomics 20:976. doi: 10.1186/s12864-019-6358-x, 31830917 PMC6909477

[ref100] Chelius M. K. Triplett E. W. (2001). The diversity of archaea and bacteria in association with the roots of Zea mays L. Microbial Ecology. 3, 252–263. 10.1007/s002480000087 11391463

[ref8] Clark E. A. Francis C. A. (1985). Transgressive yielding in bean: maize intercrops; interference in time and space. Field Crop Res. 11, 37–53. doi: 10.1016/0378-4290(85)90090-5

[ref9] Compant S. Cassan F. Kostić T. Johnson L. Brader G. Trognitz F. . (2025). Harnessing the plant microbiome for sustainable crop production. Nat. Rev. Microbiol. 23, 9–23. doi: 10.1038/s41579-024-01079-1, 39147829

[ref10] Cui J. Li S. Baoyin B. Feng Y. Guo D. Zhang L. . (2024). Maize/soybean intercropping with straw return increases crop yield by influencing the biological characteristics of soil. Microorganisms 12:1108. doi: 10.3390/microorganisms12061108, 38930490 PMC11205681

[ref11] Dang K. Gong X. Zhao G. Wang H. Ivanistau A. Feng B. (2020). Intercropping alters the soil microbial diversity and community to facilitate nitrogen assimilation: a potential mechanism for increasing proso millet grain yield. Front. Microbiol. 11:601054. doi: 10.3389/fmicb.2020.601054, 33324383 PMC7721675

[ref12] Dendooven L. Gutiérrez-Oliva V. F. Patiño-Zúñiga L. Ramírez-Villanueva D. A. Verhulst N. Luna-Guido M. . (2012). Greenhouse gas emissions under conservation agriculture compared to traditional cultivation of maize in the central highlands of Mexico. Sci. Total Environ. 431, 237–244. doi: 10.1016/j.scitotenv.2012.05.029, 22687433

[ref13] El Sabagh A. Islam M. S. Hossain A. Iqbal M. A. Mubeen M. Waleed M. . (2022). Phytohormones as growth regulators during abiotic stress tolerance in plants. Front. Agron. 4:765068. doi: 10.3389/fagro.2022.765068

[ref14] Francioli D. Kampouris I. D. Kuhl-Nagel T. Babin D. Sommermann L. Behr J. H. . (2025). Microbial inoculants modulate the rhizosphere microbiome, alleviate plant stress responses, and enhance maize growth at field scale. Genome Biol. 26:148. doi: 10.1186/s13059-025-03621-7, 40452057 PMC12128319

[ref15] Gao Y. Duan A. Qiu X. Liu Z. Sun J. Zhang J. . (2010). Distribution of roots and root length density in a maize/soybean strip intercropping system. Agric. Water Manag. 98, 199–212. doi: 10.1016/j.agwat.2010.08.021

[ref16] Gao C. Montoya L. Xu L. Madera M. Hollingsworth J. Purdom E. . (2020). Fungal community assembly in drought-stressed sorghum shows stochasticity, selection, and universal ecological dynamics. Nat. Commun. 11:34. doi: 10.1038/s41467-019-13913-9, 31911594 PMC6946711

[ref17] Good A. G. Shrawat A. K. Muench D. G. (2004). Can less yield more? Is reducing nutrient input into the environment compatible with maintaining crop production? Trends Plant Sci. 9, 597–605. doi: 10.1016/j.tplants.2004.10.008, 15564127

[ref18] Guo X. Hou Z. Wu X. Liu W. Cai J. An S. (2025). Long-term intercropping shaped soil bacterial microbiome composition and structure of maize fields in a semiarid region. Soil Tillage Res. 247:106383. doi: 10.1016/j.still.2024.106383

[ref19] Hartmann M. Six J. (2023). Soil structure and microbiome functions in agroecosystems. Nat. Rev. Earth Environ. 4, 4–18. doi: 10.1038/s43017-022-00366-w

[ref101] Horton M. W. Bodenhausen N. Beilsmith K. Meng D. Muegge B. D. Subramanian S. . (2014). Genome-wide association study of Arabidopsis thaliana leaf microbial community. Nat. Commun. 5, 5320., 25382143 10.1038/ncomms6320 PMC4232226

[ref20] Jensen E. S. Carlsson G. Hauggaard-Nielsen H. (2020). Intercropping of grain legumes and cereals improves the use of soil N resources and reduces the requirement for synthetic fertilizer N: a global-scale analysis. Agron. Sustain. Dev. 40:5. doi: 10.1007/s13593-020-0607-x

[ref21] Jiang Y. Khan M. U. Lin X. Lin Z. Lin S. Lin W. (2022). Evaluation of maize/peanut intercropping effects on microbial assembly, root exudates and peanut nitrogen uptake. Plant Physiol. Biochem. 171, 75–83. doi: 10.1016/j.plaphy.2021.12.024, 34973502

[ref22] Jiang P. Wang Y. Zhang Y. Fei J. Rong X. Peng J. . (2024a). Intercropping enhances maize growth and nutrient uptake by driving the link between rhizosphere metabolites and microbiomes. New Phytol. 243, 1506–1521. doi: 10.1111/nph.19906, 38874414

[ref23] Jiang P. Wang Y. Zhang Y. Fei J. Rong X. Peng J. . (2024b). Enhanced productivity of maize through intercropping is associated with community composition, core species, and network complexity of abundant microbiota in rhizosphere soil. Geoderma 442:116786. doi: 10.1016/j.geoderma.2024.116786

[ref24] Jing J. Cong W. Bezemer T. M. (2022). Legacies at work: plant-soil-microbiome interactions underpinning agricultural sustainability. Trends Plant Sci. 27, 781–792. doi: 10.1016/j.tplants.2022.05.007, 35701291

[ref25] Kwak M.-J. Kong H. G. Choi K. Kwon S.-K. Song J. Y. Lee J. . (2018). Rhizosphere microbiome structure alters to enable wilt resistance in tomato. Nat. Biotechnol. 36, 1100–1109. doi: 10.1038/nbt.4232, 30295674

[ref26] Labouyrie M. Ballabio C. Romero F. Panagos P. Jones A. Schmid M. W. . (2023). Patterns in soil microbial diversity across Europe. Nat. Commun. 14:3311. doi: 10.1038/s41467-023-37937-4, 37291086 PMC10250377

[ref27] Lai H. Gao F. Su H. Zheng P. Li Y. Yao H. (2022). Nitrogen distribution and soil microbial community characteristics in a legume–cereal intercropping system: a review. Agron. 12:1900. doi: 10.3390/agronomy12081900

[ref28] Lal R. (1997). Long-term tillage and maize monoculture effects on a tropical Alfisol in western Nigeria. I. Crop yield and soil physical properties. Soil Tillage Res. 42, 145–160. doi: 10.1016/S0167-1987(97)00006-8

[ref29] Li X. Mu Y. Cheng Y. Liu X. Nian H. (2012). Effects of intercropping sugarcane and soybean on growth, rhizosphere soil microbes, nitrogen and phosphorus availability. Acta Physiol. Plant. 35, 1113–1119. doi: 10.1007/s11738-012-1148-y

[ref30] Li L. Tilman D. Lambers H. Zhang F. S. (2014). Plant diversity and overyielding: insights from belowground facilitation of intercropping in agriculture. New Phytol. 203, 63–69. doi: 10.1111/nph.12778, 25013876

[ref31] Li X. Wang C. Zhu X. Ntoukakis V. Cernava T. Jin D. (2023). Exploration of phyllosphere microbiomes in wheat varieties with differing aphid resistance. Environ. Microbiome 18:78. doi: 10.1186/s40793-023-00534-5, 37876011 PMC10594911

[ref32] Lidbury I. Borsetto C. Murphy A. R. J. Bottrill A. Jones A. M. E. Bending G. D. . (2021). Niche-adaptation in plant-associated Bacteroidetes favours specialisation in organic phosphorus mineralisation. ISME J. 15, 1040–1055. doi: 10.1038/s41396-020-00829-2, 33257812 PMC8115612

[ref33] Louca S. Polz M. F. Mazel F. Albright M. B. N. Huber J. A. O’Connor M. I. . (2018). Function and functional redundancy in microbial systems. Nature Ecology Evolution 2, 936–943. doi: 10.1038/s41559-018-0519-1, 29662222

[ref34] Meng L. Zhang A. Wang F. Han X. Wang D. Li S. (2015). *Arbuscular mycorrhizal* fungi and rhizobium facilitate nitrogen uptake and transfer in soybean/maize intercropping system. Front. Plant Sci. 6:339. doi: 10.3389/fpls.2015.00339, 26029236 PMC4429567

[ref35] Nadarajah K. Rahman N. S. N. A. (2021). Plant-microbe interaction: aboveground to belowground, from the good to the bad. Int. J. Mol. Sci. 22:10388. doi: 10.3390/ijms221910388, 34638728 PMC8508622

[ref36] Nasar J. Zhao C. J. Khan R. Gul H. Gitari H. Shao Z. . (2022). Maize-soybean intercropping at optimal N fertilization increases the N uptake, N yield and N use efficiency of maize crop by regulating the N assimilatory enzymes. Front. Plant Sci. 13:1077948. doi: 10.3389/fpls.2022.1077948, 36684768 PMC9846272

[ref37] Nuss E. T. Tanumihardjo S. A. (2010). Maize: a paramount staple crop in the context of global nutrition. Compr. Rev. Food Sci. Food Saf. 9, 417–436. doi: 10.1111/j.1541-4337.2010.00117.x, 33467836

[ref38] Orellana D. Machuca D. Ibeas M. A. Estevez J. M. Poupin M. J. (2022). Plant-growth promotion by proteobacterial strains depends on the availability of phosphorus and iron in *Arabidopsis thaliana* plants. Front. Microbiol. 13:1083270. doi: 10.3389/fmicb.2022.1083270, 36583055 PMC9792790

[ref39] Pan X. Raaijmakers J. M. Carrión V. J. (2023). Importance of Bacteroidetes in host–microbe interactions and ecosystem functioning. Trends Microbiol. 31, 959–971. doi: 10.1016/j.tim.2023.03.018, 37173204

[ref40] Poorter H. Niklas K. J. Reich P. B. Oleksyn J. Poot P. Mommer L. (2012). Biomass allocation to leaves, stems and roots: meta-analyses of interspecific variation and environmental control. New Phytol. 193, 30–50. doi: 10.1111/j.1469-8137.2011.03952.x, 22085245

[ref41] Qiao M. Sun R. Wang Z. Dumack K. Xie X. Dai C. . (2024). Legume rhizodeposition promotes nitrogen fixation by soil microbiota under crop diversification. Nat. Commun. 15:2924. doi: 10.1038/s41467-024-47159-x, 38575565 PMC10995168

[ref42] Qin Y. Pan X. Yuan Z. (2016). Seed endophytic microbiota in a coastal plant and phytobeneficial properties of the fungus *Cladosporium cladosporioides*. Fungal Ecol. 24, 53–60. doi: 10.1016/j.funeco.2016.08.011

[ref43] Rastogi G. Sbodio A. Tech J. J. Suslow T. V. Coaker G. L. Leveau J. H. (2012). Leaf microbiota in an agroecosystem: spatiotemporal variation in bacterial community composition on field-grown lettuce. ISME J. 6, 1812–1822. doi: 10.1038/ismej.2012.32, 22534606 PMC3446804

[ref44] Schlechter R. O. Kear E. J. Bernach M. Remus D. M. Remus-Emsermann M. N. P. (2023). Metabolic resource overlap impacts competition among phyllosphere bacteria. ISME J. 17, 1445–1454. doi: 10.1038/s41396-023-01459-0, 37355740 PMC10432529

[ref103] Schoch C. L. Seifert K. A. Huhndorf S. Robert V. Spouge J. L. Levesque C. A. . (2012). Nuclear ribosomal internal transcribed spacer (ITS) region as a universal DNA barcode marker for Fungi. Proc. Natl. Acad. Sci. U.S.A. 109, 6241–6246. doi: 10.1073/pnas.1117018109, 22454494 PMC3341068

[ref45] Shao X. Yang C. Chen Y. Liu C. Liu C. Shi X. . (2025). Sorghum-peanut intercropping under salt stress mediates rhizosphere microbial community shaping in sorghum by affecting soil sugar metabolism pathways. Front. Microbiol. 16:1589415. doi: 10.3389/fmicb.2025.1589415, 40376464 PMC12078205

[ref46] Stern W. R. (1993). Nitrogen fixation and transfer in intercrop systems. Field Crop Res. 34, 335–356. doi: 10.1016/0378-4290(93)90121-3

[ref102] Tan X. Xie H. Yu J. Wang Y. Xu J. Xu P. . (2022). Host genetic determinants drive compartment‐specific assembly of tea plant microbiomes. Plant Biotechnology Journal. 11:2174–2186. 10.1111/pbi.13897 PMC9616527 35876474

[ref47] Tilman D. (2020). Benefits of intensive agricultural intercropping. Nat Plants 6, 604–605. doi: 10.1038/s41477-020-0677-4, 32483327

[ref48] Tilman D. Balzer C. Hill J. Befort B. L. (2011). Global food demand and the sustainable intensification of agriculture. Proc. Natl. Acad. Sci. USA 108, 20260–20264. doi: 10.1073/pnas.1116437108, 22106295 PMC3250154

[ref49] Trivedi P. Leach J. E. Tringe S. G. Sa T. Singh B. K. (2021). Plant-microbiome interactions: from community assembly to plant health. Nat. Rev. Microbiol. 19:72. doi: 10.1038/s41579-020-00490-8, 33230338

[ref50] Vorholt J. A. (2012). Microbial life in the phyllosphere. Nat. Rev. Microbiol. 10, 828–840. doi: 10.1038/nrmicro2910 23154261

[ref51] Wang C. Huang X. Yu J. Liu Y. Qu F. Wang J. . (2026). Nonlinear effect of microbial diversity loss on soil carbon flux. Soil Biol. Biochem. 213:110028. doi: 10.1016/j.soilbio.2025.110028

[ref52] Wang X. Li X. Wang Z. Long A. Ji X. Gong X. . (2025). Straw return increased maize phosphorus uptake and grain yield by alleviating rhizosphere soil microbial metabolism limitation: insights from ecoenzymatic stoichiometry. Plant Soil 515, 1781–1799. doi: 10.1007/s11104-025-07690-2

[ref53] Wang W. Li M. Zhou R. Mo F. Khan A. Batool A. . (2023). Leaf senescence, nitrogen remobilization, and productivity of maize in two semiarid intercropping systems. Eur. J. Agron. 150:126943. doi: 10.1016/j.eja.2023.126943

[ref54] Wang Y. Zhang Y. Yang Z. Fei J. Zhou X. Rong X. . (2024). Intercropping improves maize yield and nitrogen uptake by regulating nitrogen transformation and functional microbial abundance in rhizosphere soil. J. Environ. Manag. 358:120886. doi: 10.1016/j.jenvman.2024.120886, 38648726

[ref55] Wicaksono W. A. Köberl M. White R. A. Jansson J. K. Jansson C. Cernava T. . (2024). Plant-specific microbial diversity facilitates functional redundancy at the soil-root interface. Plant Soil, 1–15. doi: 10.1007/s11104-024-07097-5 PMC13337600 42416386

[ref56] Xia H. Zhao J. Sun J. Bao X. Christie P. Zhang F. . (2013). Dynamics of root length and distribution and shoot biomass of maize as affected by intercropping with different companion crops and phosphorus application rates. Field Crop Res. 150, 52–62. doi: 10.1016/j.fcr.2013.05.027

[ref57] Xie X. Liu Y. Chen G. Turatsinze A. N. Yue L. Ye A. . (2024). Granular bacterial inoculant alters the rhizosphere microbiome and soil aggregate fractionation to affect phosphorus fractions and maize growth. Sci. Total Environ. 912:169371. doi: 10.1016/j.scitotenv.2023.169371, 38104809

[ref58] Xiong C. Singh B. K. He J. Han Y. Li P. Wan L. . (2021a). Plant developmental stage drives the differentiation in ecological role of the maize microbiome. Microbiome 9:171. doi: 10.1186/s40168-021-01118-6, 34389047 PMC8364065

[ref59] Xiong C. Zhu Y.-G. Wang J.-T. Singh B. Han L.-L. Shen J.-P. . (2021b). Host selection shapes crop microbiome assembly and network complexity. New Phytol. 229, 1091–1104. doi: 10.1111/nph.16890 32852792

[ref60] You L. Ros G. H. Chen Y. Shao Q. Young M. D. Zhang F. . (2023). Global mean nitrogen recovery efficiency in croplands can be enhanced by optimal nutrient, crop and soil management practices. Nat. Commun. 14:5747. doi: 10.1038/s41467-023-41504-2, 37717014 PMC10505174

[ref61] Zhang L. Feng Y. Zhao Z. Cui Z. Baoyin B. Wang H. . (2024). Maize/soybean intercropping with nitrogen supply levels increases maize yield and nitrogen uptake by influencing the rhizosphere bacterial diversity of soil. Front. Plant Sci. 15:1437631. doi: 10.3389/fpls.2024.1437631, 39290744 PMC11405324

[ref62] Zhang J. Liu Y. X. Zhang N. Hu B. Jin T. Xu H. . (2019). NRT1.1B is associated with root microbiota composition and nitrogen use in field-grown rice. Nat. Biotechnol. 37, 676–684. doi: 10.1038/s41587-019-0104-4, 31036930

[ref63] Zhang R. Mu Y. Li X. Li S. Sang P. Wang X. . (2020). Response of the *Arbuscular mycorrhizal* fungi diversity and community in maize and soybean rhizosphere soil and roots to intercropping systems with different nitrogen application rates. Sci. Total Environ. 740:139810. doi: 10.1016/j.scitotenv.2020.139810, 32563865

[ref64] Zhao X. Dong Q. Han Y. Zhang K. Shi X. Yang X. . (2022). Maize/peanut intercropping improves nutrient uptake of side-row maize and system microbial community diversity. BMC Microbiol. 22:14. doi: 10.1186/s12866-021-02425-6, 34996375 PMC8740425

[ref65] Zheng B. Zhang X. Chen P. Du Q. Zhou Y. Yang H. . (2021). Improving maize’s N uptake and N use efficiency by strengthening roots’ absorption capacity when intercropped with legumes. PeerJ 9:e11658. doi: 10.7717/peerj.11658, 34221735 PMC8234926

