# Supplementary material for: Niche-specific maize microbiomes enhance productivity and nitrogen uptake under intercropping
Source: Front Microbiol. 2026 Jan 15;16:1711988. doi: 10.3389/fmicb.2025.1711988 (PMC12852346; doi:10.3389/fmicb.2025.1711988)
Supplement: Supplementary file 1 [file Data_Sheet_1.DOCX]

**Niche-specific maize microbiomes enhance productivity and nitrogen uptake under intercropping**

Yao Chang^1^, Jian Wang^2,^ *, Chengbin Xu^1^, Fangying Qu^2, 3^, Xuekai Sun^2^, Zhi Quan^2^, Liming Yin^2^, Yunting Fang^2^, Chao Wang^2,^ *

^1^School of Environmental Science, Liaoning University, Shenyang 110036, China.

^2^CAS Key Laboratory of Forest Ecology and Silviculture, Institute of Applied Ecology. Chinese Academy of Sciences, Shenyang, 110016, China.

^3^University of Chinese Academy of Sciences, Beijing, 100049, China.

***Corresponding author:**

Chao Wang & Jian Wang

Institute of Applied Ecology, Chinese Academy of Sciences

No. 72 Wenhua Road, Shenyang, Liaoning, 110016, China

Telephone: +86-24-83970570

Email: [cwang@iae.ac.cn](mailto:cwang@iae.ac.cn) & [wj@iae.ac.cn](mailto:wj@iae.ac.cn)

**This file contains:**

**Figure S1 The experiment followed a randomized block design with two treatments: maize monoculture (MM) and maize-soybean intercropping (IM).** Each treatment included three biological replicates (n = 3) arranged in 40 × 40 m^2^ plots. In the IM system, six maize rows alternated with four soybean rows (6M:4S). Maize was planted with 23 cm in-row spacing and 58 cm between rows, while soybean was planted with 15 cm in-row spacing and 65 cm between rows.

**Figure S2 Profiles of the major predicted functional potentials of bacterial and fungal communities across aboveground and belowground maize niches under monoculture (MM) and intercropping (IM).** Displayed functions represent the dominant predicted functional categories within each community. Panels illustrate (A) belowground bacterial functions, (B) aboveground bacterial functions, (C) belowground fungal functions, and (D) aboveground fungal functions. Aboveground niches include the phylloplane, leaf endosphere (Leaf en), stem episphere (Stem ep), and stem endosphere (Stem en), while belowground niches include the rhizoplane, root endosphere (Root en), bulk soil, and rhizosphere soil.


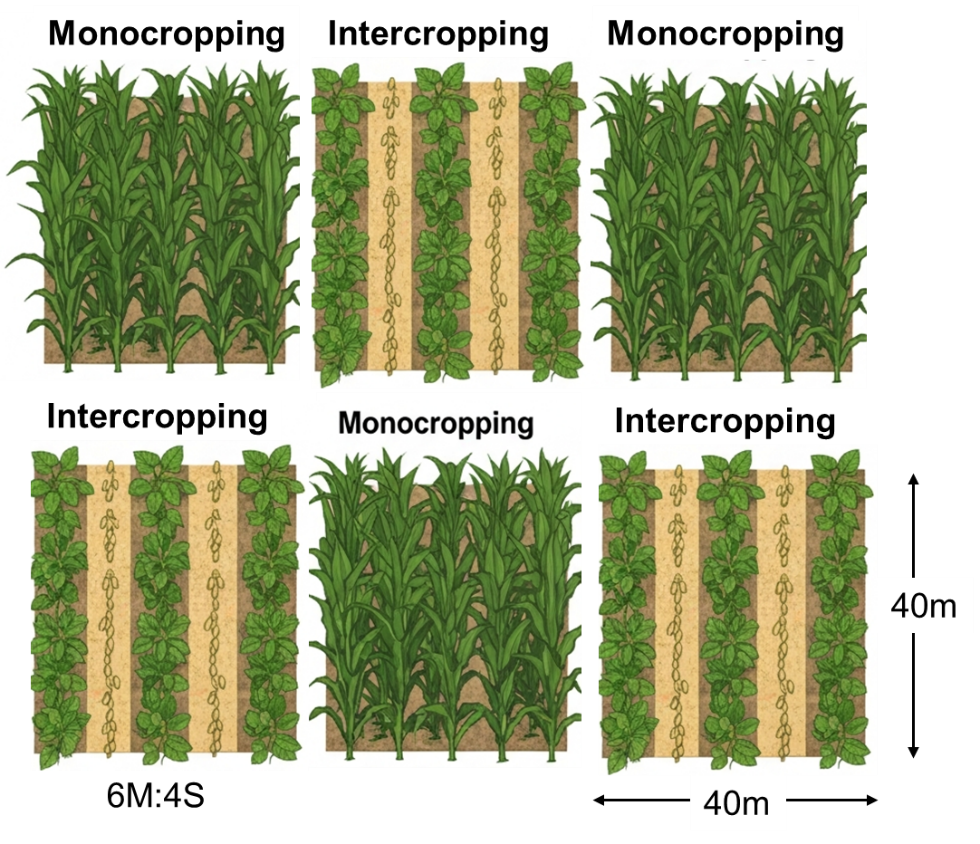


**Figure S1**


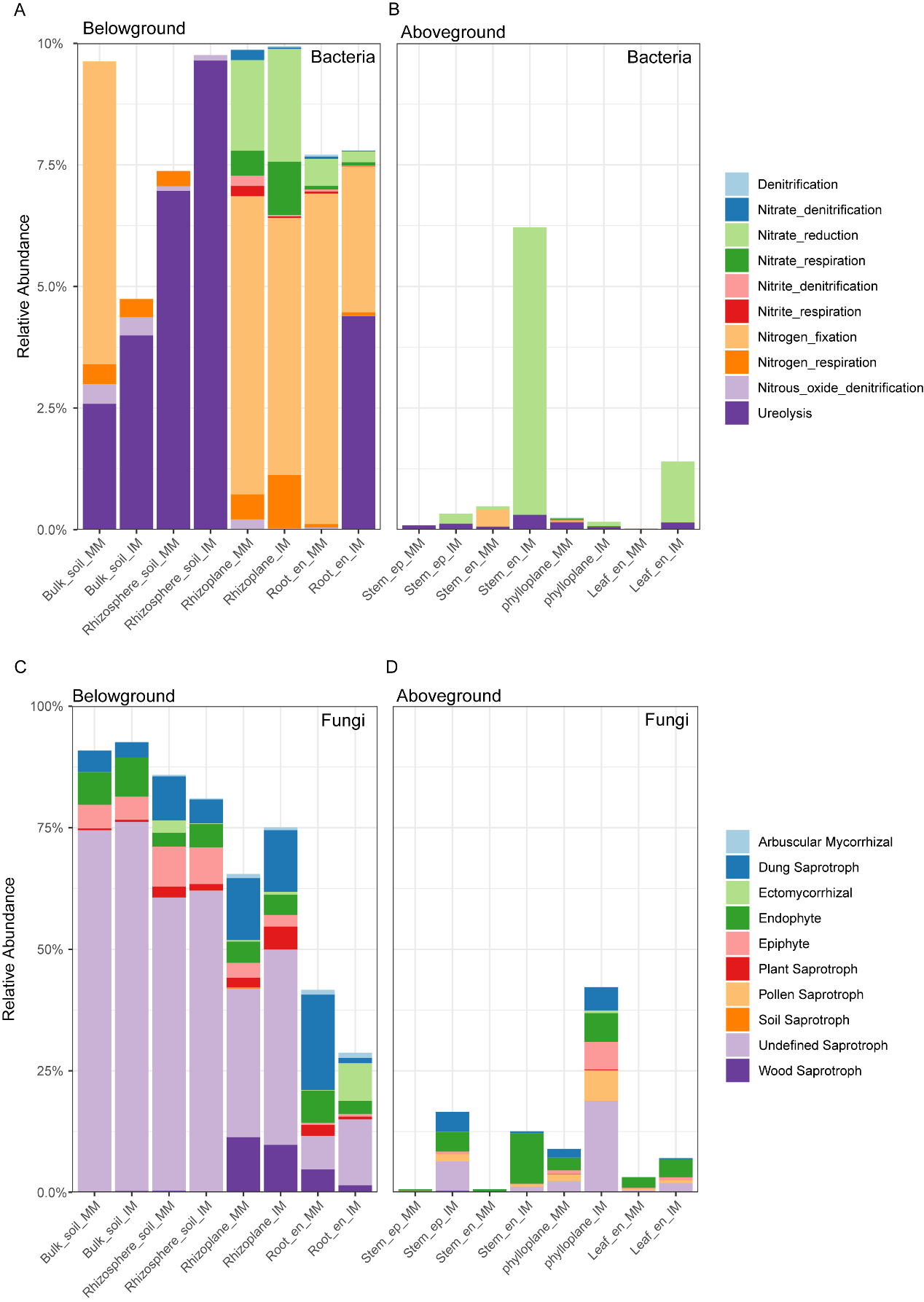


**Figure S2**
